# Séance: reference-based phylogenetic analysis for 18S rRNA studies

**DOI:** 10.1186/s12862-014-0235-7

**Published:** 2014-11-30

**Authors:** Alan Medlar, Tuomas Aivelo, Ari Löytynoja

**Affiliations:** Institute of Biotechnology, University of Helsinki, Helsinki, P.O.Box 56 Finland

**Keywords:** rRNA marker genes, 18S community analysis, Phylogenetic placement

## Abstract

**Background:**

Marker gene studies often use short amplicons spanning one or more hypervariable regions from an rRNA gene to interrogate the community structure of uncultured environmental samples. Target regions are chosen for their discriminatory power, but the limited phylogenetic signal of short high-throughput sequencing reads precludes accurate phylogenetic analysis. This is particularly unfortunate in the study of microscopic eukaryotes where horizontal gene flow is limited and the rRNA gene is expected to accurately reflect the species phylogeny. A promising alternative to full phylogenetic analysis is phylogenetic placement, where a reference phylogeny is inferred using the complete marker gene and iteratively extended with the short sequences from a metagenetic sample under study.

**Results:**

Based on the phylogenetic placement approach we built Séance, a community analysis pipeline focused on the analysis of 18S marker gene data. Séance combines the alignment extension and phylogenetic placement capabilities of the Pagan multiple sequence alignment program with a suite of tools to preprocess, cluster and visualise datasets composed of many samples. We showcase Séance by analysing 454 data from a longitudinal study of intestinal parasite communities in wild rufous mouse lemurs (*Microcebus rufus*) as well as in simulation. We demonstrate both improved OTU picking at higher levels of sequence similarity for 454 data and show the accuracy of phylogenetic placement to be comparable to maximum likelihood methods for lower numbers of taxa.

**Conclusions:**

Séance is an open source community analysis pipeline that provides reference-based phylogenetic analysis for rRNA marker gene studies. Whilst in this article we focus on studying nematodes using the 18S marker gene, the concepts are generic and reference data for alternative marker genes can be easily created. Séance can be downloaded from http://wasabiapp.org/software/seance/.

**Electronic supplementary material:**

The online version of this article (doi:10.1186/s12862-014-0235-7) contains supplementary material, which is available to authorized users.

## Background

Ribosomal RNA (rRNA) marker gene studies remain central in the characterisation of the community structure of uncultured microbes and microscopic eukaryotes in environmental samples. The introduction of high-throughput sequencing has increased the resolution of rRNA studies. However, in exchange for high volumes of data, modern sequencing platforms exhibit higher error rates and produce shorter reads than traditional sequencers. When sequencing a single organism, large quantities of data are used to overcome errors, for example, using high coverage in resequencing [1,2] and *k*-mer-based error correction in *de-novo* assembly [3]. In the case of community analysis, where species diversity is estimated *in-situ*, each read could be representative of a unique species or be completely erroneous. Failure to account for these issues can result in inflated estimations of species diversity and the errors propagated to subsequent analyses [4].

Methodological advances have focused on improving our ability to accurately estimate the level of species diversity given the large number of potential confounders, for example, appropriate techniques for read filtering and quality trimming [5], the identification and removal of artefacts from amplification [6] and pyrosequencing [7,8] and improved methods for clustering reads into operational taxonomic units (OTUs) [9]. Many of these advances have been incorporated into integrated pipelines [10,11] and packaged along with traditional phylogenetic analysis tools. Phylogenetics is used to classify sequences of unknown origin based on their evolutionary relationships to all other sequences being considered. The phylogenetic context of a sequence is only reliant on the information content of the actual sequence data and is robust against issues of incompleteness that plague similarity-based classification techniques. Unfortunately, while phylogenetic analysis can be appropriate for data from older sequencing technologies, the limited information content of short amplicon sequences constrains our ability to make accurate inferences, leading to topological errors and inappropriate branch lengths.

A recently developed alternative [12,13] to full phylogenetic analysis is to infer the evolutionary relationships between known species *a priori* using the full length marker gene and place the shorter sequences from our samples into that reference tree. So-called phylogenetic placement has many advantages: (i) the tree produced is expected to be more accurate as the overall structure of the tree is based on many more sequences and a greater number of informative sites per sequence, (ii) it is computationally efficient as sequences are placed with respect to a fixed tree topology with fixed branch lengths and (iii) it insulates the user from error as the task of constructing the initial reference tree is effectively outsourced to an expert. One such tool to embody this approach is Pagan [14], a phylogeny-aware multiple sequence alignment method capable of extending a reference alignment by placing a query sequence into its correct phylogenetic context. Pagan uses partial-order sequence graphs [15] to model non-linear dependencies between characters (indels) and uncertainties in the input data (putative homopolymer errors). Pagan has been shown to perform well in the phylogenetic placement problem due to maintaining all the information from the original reference sequences and inferred ancestral sequences in this graphical format [14].

In order to take advantage of Pagan’s capabilities in the field of metagenetics, we built Séance, a community analysis pipeline providing a comprehensive framework integrating phylogenetic placement with several other tools for amplicon-based analyses. The core contributions of this work are:

- We provide a complete bioinformatics pipeline capable of handling raw data from both 454/Roche and Illumina platforms and that performs the necessary quality checks, read filtering, denoising and OTU clustering for further analysis.
- We explore the efficacy of using Pagan for amplicon-based marker gene studies, using the modelling of homopolymer errors in clustering and phylogenetic placement of cluster centroid sequences into a reference tree for phylogenetic analysis.
- Séance is open source software and makes use of the standardised biological observation matrix (BIOM) file format [16] to allow easy integration with Mothur [10], QIIME [11] and other conforming pipelines.

## Implementation

**Figure 1:**
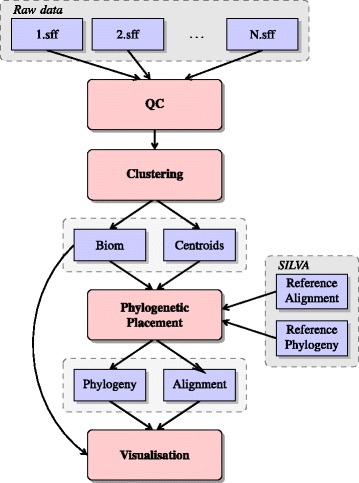
**Séance workflow overview.** Overview of the complete Séance workflow. Input files can be either raw SFF files from Roche/454 or FASTQ files from other platforms. The clustering command outputs a BIOM file and cluster centroids in a FASTA file. The phylogenetic placement command outputs a phylogeny and a phylogenetically consistent multiple sequence alignment.

A high-level overview of Séance’s workflow is shown in Figure 1. Séance’s capabilities are split into four submodules: preprocessing (QC), clustering, phylogenetic placement and visualisation.

### Preprocessing

The Séance pipeline accepts either FASTQ files or can work directly with raw SFF data containing 454 amplicon sequences. Séance provides support for preprocessing by building on many other tools, e.g. denoising of 454 amplicons is performed by Ampliconnoise [7] and chimera discovery with UCHIME [6]. Séance performs additional filtering based on quality scores (minimum, average and windowed), ambiguous base calls, sequence length and the number of errors in barcode and primer sequences. The preprocessing step also includes dereplication, trimming of barcodes and primers, and truncating sequences to a uniform length.

### Clustering

**Figure 2:**
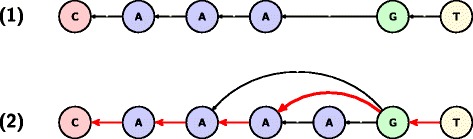
**Homopolymer modelling.** The cluster centroid **(1)** is higher in abundance than the query sequence **(2)**. When the query is aligned with the centroid sequence, the alignment score is maximised by the dynamic programming algorithm traversing the red path through the sequence graph defined by the query sequence and Pagan’s homopolymer heuristics.

Séance constructs OTUs by greedily clustering reads in descending order of abundance. Our approach is based on the assumption that the sequences found in highest abundance are error-free and those reads containing PCR or sequencing artefacts are not only lower in abundance but highly similar to the error-free sequences they originated from. Taking abundance into consideration during clustering is similar to many other approaches [9,17]. Séance differs from these approaches by using Pagan’s modelling of homopolymer length uncertainty on sequences from platforms known to produce such errors (e.g. Roche/454). This modelling reduces alignment errors around low-complexity regions separated by short spacers and affects the calculation of sequence similarity. Figure 2 illustrates this approach by showing how skipping the last base from the homopolymer AAAA maximises the alignment score and that this gap should not contribute to the similarity calculation.

Even for samples that have been denoised, modelling of homopolymers is still useful. Denoising is performed on a per-sample basis and low abundance sequences may still contain errors. As clustering is performed across all samples, homopolymer modelling aims to prevent these sequences from being erroneously assigned as new clusters.

Séance provides several options for affecting what sequences are used as an input to clustering and what clusters are output. Users can choose to only cluster sequences with a copy-number greater than *n* in a given sample. This can be used to remove singleton sequences (more likely to be erroneous) or to reduce the computational burden of clustering. Users can additionally output only those clusters that contain sequences from multiple samples. Limiting output clusters in this manner has several uses in situations where individuals are sampled multiple times or a large number of samples are analysed. For example, an OTU appearing only once may be considered irrelevant from the perspective of a specific analysis, such as the identification of temporal patterns.

### Phylogenetic placement

Séance does not infer a phylogenetic tree directly from the cluster centroid sequences but rather uses Pagan’s phylogenetic placement capabilities to insert these sequences into a fixed reference tree. To maximise accuracy, we suggest that the reference tree is built using the entire marker gene from a representative sample of the clade containing the target species.

In brief, placing a query sequence of unknown origin with Pagan involves: (i) searching for the optimal placement among the set of target nodes (extant or ancestral nodes in the reference phylogeny), (ii) aligning the query against the (observed or inferred) sequence at the best target, and (iii) adding the query both in the reference tree (a new branch) and the reference alignment (a new sequence), adjusting the latter for any insertions in the new sequence. Pagan can speed up the target search using an external local aligner and allows for considering only a subset of target nodes. In our current implementation of the Séance pipeline, we only permit query sequences to be aligned against extant sequences (i.e. we assume that the query sequences or their close relatives are in the reference data) but Pagan can also consider inferred ancestral sequences and add a query sequence as a new evolutionary lineage in the reference phylogeny.

The final output tree is pruned to contain only the placed query sequences and those nodes from the reference tree necessary to ensure a minimum spanning tree. By default, Pagan produces an alignment of the full reference data and the newly added query sequences but it can also prune the output; for practical purposes Séance outputs an alignment trimmed around the target region and only contains the query sequences together with their closest reference sequences.

### Visual inspection and graphics

Séance provides several options for visualisation. Cluster centroids can be visually inspected with Wasabi, a web application integrating evolutionary alignment tools and interactive visualisation (unpublished, http://www.wasabiapp.org). Wasabi permits the user to see the sequence alignment resulting from phylogenetic placement comparing how cluster centroids match the closest reference sequence.

**Figure 3:**
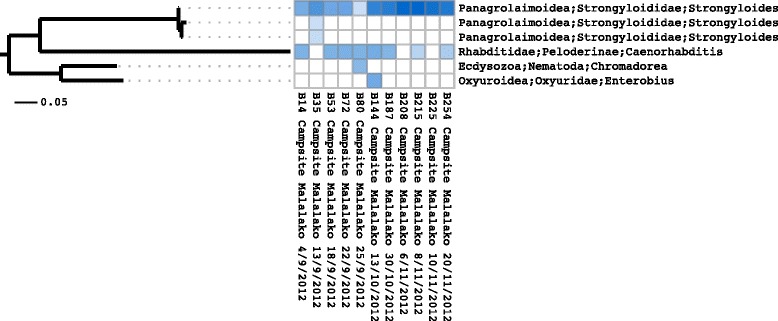
**Heatmap and phylogenetic placement derived tree.** Heatmap showing relative abundances of OTUs together with corresponding phylogenetic tree generated with phylogenetic placement of OTU centroid sequences into complete marker gene alignment and tree. This figure was generated using the Séance ‘heatmap’ command.

For publication figures, phylogenetic trees and cluster abundances are combined in a single heatmap. An example is shown in Figure 3. Figures can be generated for the complete dataset or a subset of samples.

## Results

We used Séance to analyse all of the samples from a longitudinal study of intestinal parasite communities in wild rufous mouse lemurs (*Microcebus rufus*) from Ranomafana National Park in southeast Madagascar (Aivelo T, Medlar A, Löytynoja A, Laakkonen J, Jernvall J: Tracking year–to–year changes in intestinal nematode communities, in preparation) The raw sequence data has been submitted to the Short Read Archive under project number SRP042187. Faecal samples containing eggs were collected and analysed as a proxy for the actual parasite communities in mouse lemur intestines. High levels of contamination were expected as flies can lay eggs in the faeces during the time between trapping and collection.

The dataset is composed of 324 samples collected during a three year period. A majority of samples are from lemurs that were sampled at multiple time points throughout the collection period. A 450 base pair region within the 18S small subunit rRNA gene was amplified containing the V5 hypervariable region and sequenced using a Roche/454 Genome Sequencer FLX. Each sample was denoised using Ampliconnoise (ver. 1.29). Sequences with ambiguous base calls, more than one error in the barcode or more than two errors in the primer sequence were discarded. Barcodes and primers were removed and all sequences truncated to 250bp. Putative chimeric sequences were removed using UCHIME (ver. 4.2.40) in *de novo* mode. Preprocessing all 324 samples resulted in 511,687 high-quality reads comprised of 10,369 unique sequences.

Here we focused on the impact of Séance’s use of Pagan’s homopolymer modelling on identification of OTUs and phylogenetic placement of centroid sequences into a reference tree.

### Homopolymer modelling impacts OTU identification

As an input to clustering we excluded sequences with a copy number less than 5. This step filters out all but 443 of the 10,369 unique sequences we started with. Due to dereplication, however, these 443 sequences were representative of 97.1% of the total reads that passed quality control. We expect that a great majority of excluded sequences represent unfiltered PCR artefacts and sequencing errors. Furthermore, even the true variants potentially lost are either very rare or present in one sample only and therefore uninformative in a longitudinal study focusing on community evolution.

Clustering at 99% similarity with homopolymer modelling yielded 102 OTUs but without homopolymer modelling, that resulted in 132 OTUs. A MegaBLAST [18] search of the NCBI NR (non-redundant) database was performed using the centroid sequence of each cluster and a taxonomic label was derived from the lowest common ancestor of the top scoring BLAST hits from the NCBI taxonomy. Comparing taxonomic labels from the two approaches, the OTUs produced using homopolymer modelling are a subset of the OTUs produced without. Of the 30 OTUs that made up the difference, 28 were low abundance duplicates of other OTUs (for example, homopolymer modelling avoided the creation of 10 duplicate *Strongyloides* OTUs) and 2 OTUs were missed by the homopolymer modelling approach. These unique OTUs, however, appear suspicious as they are both composed of very few reads and were present in only one or two samples.

As expected, modelling of homopolymers is less important as the similarity threshold for clustering is reduced. Clustering the same set of sequences at 97% similarity yielded 52 and 58 OTUs when using and not using homopolymer modelling, respectively.

### Phylogenetic placement reduces topological errors

#### Case study

**Figure 4:**
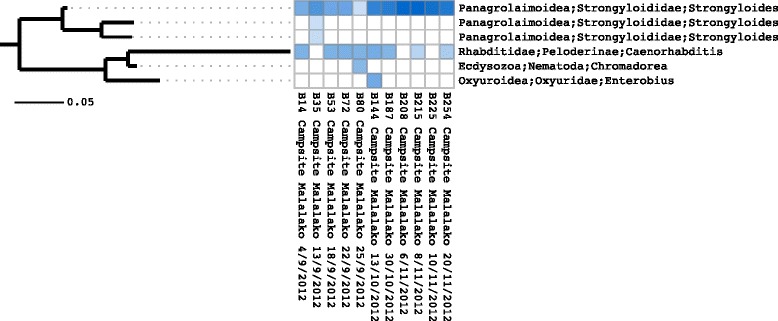
**Heatmap with** ***de novo*** **phylogenetic tree.** Heatmap showing relative abundances of OTUs together with corresponding phylogenetic tree inferred using just OTU centroid sequences. This figure was generated using the Séance ‘heatmap’ command.

To build a reference phylogenetic tree, we extracted the complete 18S rRNA alignment from 1320 members of the phylum Nematoda from the SILVA database (SSURef NR 115) [19]. After the removal of columns which contained only gaps, a tree was inferred using RAxML (ver. 7.2.8) [20]. RAxML was run with the GTR +*Γ* substitution model for 10 repetitions. We are aware that the SILVA alignment is not perfect and alignment errors may adversely affect results but the use of the original alignment allows for reproducibility. Next, we used Séance’s phylogenetic placement command to place the cluster centroid sequences into the reference tree with Pagan. Figure 3 shows the result but for the purposes of exposition we have limited it to only those OTUs that appear in the data for a lemur called Malalako. For comparison, we aligned the cluster centroid sequences using MAFFT (ver. 7.149b) [21] and inferred a tree, *de novo*, using RAxML. The tree was manually rerooted and is shown in Figure 4. Whilst the two phylogenetic trees are very similar, in this example there is a single topological error in the *de novo* tree in the location of the *Caenorhabditis* cluster, which should be more closely related to *Strongyloides*. We further note that the branch lengths have been underestimated in the *de novo* phylogeny due to the lower relative proportion of variable to conserved sequence compared to the complete marker gene.

#### Simulation

The Ranomafana National Park study only presents anecdotal evidence of the errors Séance can avoid thanks to using a phylogenetic placement strategy. We additionally used simulation to better understand how Séance performs, not just against *de novo* phylogenetic inference, but also against maximum likelihood-based placement methods. The methods chosen for comparison are representative of current strategies employed by other community analysis pipelines. Both Mothur and QIIME focus on microbial analysis and so, due to the potential of large numbers of OTUs, perform phylogenetic analysis using neighbour-joining or distance methods. As we were not simulating large numbers of sequences, we used RAxML which has been shown to outperform all other methods in terms of topological accuracy [22]. We used Pplacer (ver. 1.1.alpha16) [23] as an example of a phylogenetic placement method based on maximum likelihood. Pplacer has been shown to outperform other placement methods and is integrated into numerous analysis pipelines, e.g. PhyloSift [24] in the analysis of metagenomes and SEPP [25] for phylogenetic placement in exceptionally large phylogenetic trees. As input to Pplacer we use alignments from both HMMER [26] (ver. 3.1b1) and Pagan (ver. 0.55).

The simulations proceeded as follows: using the reference tree we built for the Ranomafana National Park study, we randomly selected 750 sequences to serve as a new reference tree. From those sequences not used in the reference, we selected all nematodes that would be amplified by our primer and extracted a 250bp region. We then selected *n* of these short sequences (where *n* ranged from 25–150) and used this as the input data for *de novo* tree construction (again using MAFFT and RAxML) and as query sequences for phylogenetic placement in the new reference tree using Pagan and Pplacer. The normalised Robinson-Foulds metric was calculated for both the *de novo* and phylogenetic placement-derived trees versus the minimum spanning tree for the query sequences from the original reference tree (for Pplacer, we generated a single tree from the placed sequences using the guppy utility distributed with Pplacer). For the purpose of this experiment, we assumed that the tree generated from the complete 18S gene is correct and that the trees for the target region and the full gene are concordant, though we are, of course, aware that there is no guarantee for this. For each value of *n*, we performed 1000 replicates, generating a new reference tree for each replicate. Driver scripts for running these experiments can be found in Additional file 1.

**Figure 5:**
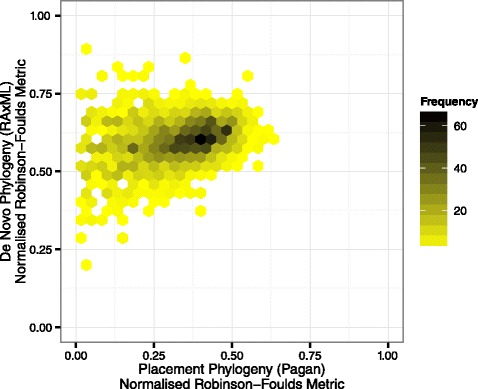
**Phylogenetic placement versus** ***de novo*** **phylogeny heatmap.** Comparison of normalised Robinson-Foulds metric of different phylogenetic methods performed on short 250bp sequences compared to the ML tree inferred from the complete marker gene: phylogenetic placement of short query sequences produces trees with fewer errors than *de novo* phylogenetic inference.

**Figure 6:**
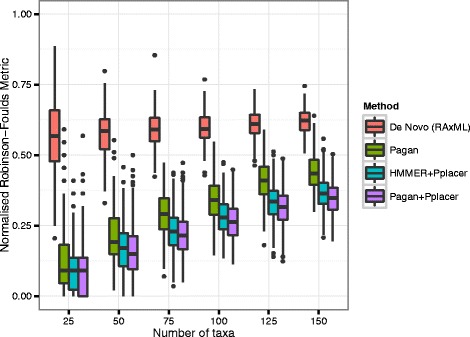
**Phylogenetic placement methods versus** ***de novo*** **phylogeny box plots.** For *de novo* phylogenies the variability of errors narrows with number of query sequences, whereas phylogenetic placement errors increase additively with the number of sequences.

Figure 5 shows a heatmap of how the accuracy of Pagan’s phylogenetic placement and *de novo* phylogenetic inference compared over all experiments. Figure 6 shows box plots of the same data broken down by the number of query sequences and include the results from HMMER+Pplacer and Pagan+Pplacer. In general, phylogenetic placement outperformed *de novo* phylogenetic inference in reconstructing the original tree due to the short sequences containing less phylogenetic information than the complete gene sequence.

For all methods of phylogenetic placement, the number of topological errors increased additively with the number of query sequences. Errors build up due to the limited information in short amplicon sequences and are made worse as our experiment only selected query sequences not present in the reference tree. This handicap will not necessarily exist in real life.

For lower numbers of query sequences, there was a small improvement using HMMER+Pplacer over Pagan, but at higher numbers of taxa, the difference became substantial. Using Pplacer together with Pagan, however, on average produced the best results, but at low numbers of taxa the improvement over using just Pagan was marginal.

## Discussion

Modelling homopolymer uncertainty during clustering allows Séance to produce fewer low abundance OTUs with duplicate labels. The process of denoising is reliant on abundance information from within a given sample, using high abundance (error-free) reads to correct low abundance ones. It follows that sequences only found in low abundance may not be denoised successfully if they contain errors [27]. As clustering is performed across all samples, homopolymer modelling aims to prevent these sequences from being erroneously assigned as new clusters by correctly matching them to existing clusters from other samples. We found this feature to be particularly important when dealing with a large number of samples as they often shared nematode species but in widely varying amounts.

Séance uses phylogenetic placement as a proxy for phylogenetic inference on the complete marker gene. *De novo* inference on the same data infers the tree given the distinct evolutionary processes acting on a single variable region which may not be concordant with the evolutionary history of the complete marker gene. Whilst, *de novo* inference is not incorrect, we argue that an approximation of the phylogenetic history of the gene under study is closer to the experimentalists intentions with the selection of a specific marker gene.

The performance of Séance’s phylogenetic placement capabilities is obviously dependent on which program is used. Our chosen method, Pagan, was outperformed by Pplacer for larger numbers of taxa. We believe this deficiency is largely due to the simple placement strategy employed by Pagan, which uses a similarity-based criterion to place sequences in the order they appear in the input file. Though we believe Pagan’s performance can be improved, other non-functional arguments lead to our choice. Pplacer can only extend phylogenies produced with maximum likelihood-based programs, but Pagan does not have this limitation.

Whilst less phylogenetic information is required to place sequences than infer a tree, Séance’s accuracy suffers if there is limited resolution in the selected variable region resulting in multiple candidate nodes for placement. In general, we have to assume that the reference data is always incomplete and therefore placing a sequence at all nodes meeting some criterion (the default behaviour of Pplacer) can be misleading as we are only able to assess uncertainty in relation to known reference data. Faced with the situation of multiple placement candidates, Séance will place the query sequence randomly on a single candidate node. The rationale is that such a placement is not wrong, it just does not communicate whether the placement is uniquely appropriate for that sequence. Ambiguous placements will generally be phylogenetically proximate and where they are not, this uncertainty is indicated in the cluster labels. We would like to emphasise that this is a design choice we made for Séance and that Pagan implements several placement strategies. If a user disagrees with this choice, they can rerun Pagan using an alternative strategy or use the multiple sequence alignment as input to Pplacer.

Future work includes supporting newer sequencing technologies and understanding how the approaches described here can be exploited with different data. In addition, Séance is currently implemented sequentially, so additional work is required to support multi-threading and computer clusters without adversely affecting results.

## Conclusions

We have presented Séance, a new software package for high-throughput amplicon community analysis. Séance exploits features from the Pagan multiple sequence alignment program to provide support for modelling homopolymer uncertainty when clustering sequences into OTUs and for phylogenetic placement of cluster centroid sequences into a reference tree. The reference files currently distributed with Séance are specific to the study of nematodes using the 18S marker gene, however, the concepts are generic and a new reference for a different target organism and marker gene can easily be created. To ease adoption of Séance, we use the BIOM file format for cluster abundance data and output cluster centroids sequences in FASTA format, this enables users to perform preprocessing and OTU clustering in a different pipeline (e.g. QIIME, Mothur), export the results and start running the Séance pipeline from the phylogenetic placement step.

## Availability and requirements

- **Project name:** Séance
- **Project home page:**http://wasabiapp.org/software/seance/
- **Operating systems:** Linux, Mac OS
- **Programming language:** Python
- **Other requirements:** External programs (see http://wasabiapp.org/software/seance/installing-seance/for complete list and instructions)
- **License:** GNU GPL v.3
- **Any restrictions to use by non-academics:** None

## Additional file

Additional file 1
**Archive of scripts and data files used in phylogenetic placement experiments.** This file can also be found at http://wasabiapp.org/download/seance/bmc_eb_seance_supplements.tar.gz
